# Ambient Backscattering-Enabled SWIPT Relaying System with a Nonlinear Energy Harvesting Model

**DOI:** 10.3390/s20164534

**Published:** 2020-08-13

**Authors:** Thu L. N. Nguyen, Jin-Young Kim, Yoan Shin

**Affiliations:** 1 School of Electronic Engineering, Soongsil University, Seoul 06978, Korea; thunguyen@ssu.ac.kr; 2 Department of Wireless Communications Engineering, Kwangwoon University, Seoul 01897, Korea; jinyoung@kw.ac.kr

**Keywords:** simultaneous wireless information and power transfer, ambient backscatter communication, nonlinear energy harvesting model, decode-and-forward relay

## Abstract

Since radio frequency (RF) signals can be used for both information transmission and energy harvesting, RF-based energy harvesting is capable of integrating with other existing communication techniques for providing better rate–energy tradeoff and quality-of-service. Within the context of an RF-based energy harvesting relaying network, a relay node not only acts as an intermediate node to help the delivery from source to destination, but also harvests energy from an RF dedicated source to prolong its lifetime. Thus, it brings diversity gain and coverage extension as well as provides extra energy for data transmission. This paper investigates a system that enables ambient backscattering communication-assisted simultaneous wireless information and power transfer at the relay. In the proposed system, a backscatter device plays a role as a relay to meet sustainable network coverage and to harvest ambient energy as well. With a power splitting (PS) scheme, we first investigate a nonlinear energy harvesting model at the relay node. In order to adapt to the channel gains, a dynamic PS ratio is required to perform well in changing environments. Moreover, we derive mathematical expressions for the outage probability and the achievable system throughput. Numerical results show the effects of various system parameters on the outage probability and the system throughput performance.

## 1. Introduction

Energy harvesting (EH) has been known as a promising solution for overcoming energy crisis in the future [1,2,3]. Especially, the EH communication is suitable for energy-constrained wireless sensor networks because typical sensor nodes are designed for low power and low data rate scenarios. A radio frequency (RF) energy harvester converts dedicated RF power to electricity, thus, it can be used as a power supply of devices with low power consumption. With renewable energy, the RF-EH offers various benefits such as clean and cheap energy, while prolongs the network operation time of the traditional battery. Compared to other energy sources (e.g., vibration, solar/light, electromagnetic, to name a few), RF energy sources can be controlled by communication systems, which are more controllable and partially predictable because their behavior is not fully deterministic. For instance, a wireless device harvests energy from ambient RF signals from certain time instants and frequencies. In fact, RF signals not only provide a suitable energy source for EH wireless communications, but also carry information. Hence, it is possible to process the same RF signals simultaneously for both energy charging and information decoding.

Different from the conventional approaches such as radio frequency identification (RFID), backscatter communication (BC) exploits a concept of modulating information on the reflected RF signals by adjusting antenna impedance, thus enables a reduction of energy consumption [2,3]. According to the capability of the backscattered devices, we classify the BC systems into three categories: monostatic backscatter, bistatic backscatter, and ambient backscatter. Among them, a device using ambient backscatter communication (AmBC) usually exploits the surrounding RF signals to enable passive devices to communicate with each other [4,5,6,7]. One example is the RF-based EH which can be generated and captured from either intentional/dedicated RF sources or ambient RF sources. With a fixed network, the harvested energy is predictable and relatively stable over time due to fixed distance. The amount of harvested energy depends on the available power density of the RF source, the communication distance, and the propagation model. For instance, the available power density of an ambient signal (e.g., microwatt-level) is much lower than near-field dedicated one (up to watt-level) [4,5]. Hence, such ambient RF sources are mainly suitable for low-power networks that enable internet-of-things (IoT) applications. From the hardware point of view, an AmBC node acts like a passive repeater by changing its reflection coefficient. A simple AmBC circuit generates binary coefficients by switching its antenna load among two states: non-reflection and reflection. In the non-reflection stage, the AmBC antenna absorbs the surrounding RF signals by energy harvester, while in the reflection state, the antenna reflects the received RF signals. One major advantage of AmBC is energy efficiency enhancement in many aspects. First, ambient RF resources are often available from anywhere in the surrounding environments, which can be utilized for AmBC. Second, it avoids the problem of radio signal generation that consumes much power in radio communication. Third, with modern EH techniques AmBC hardware become smaller, battery-free and have lower costs. In summary, the AmBC enables lower power connectivity, accumulates and preserves the energy to innovate green communications when deploying IoT-based services for the next generation.

Generally, since RF signals carry both energy and information at the same time, simultaneous information and power transfer (SWIPT) can be performed from the same input signal. The authors in [3] presented several practical receiver designs which could decode information and harvest energy with the same antenna. In SWIPT, the receiver splits the received signals into two parts by either time splitting (TS) or power splitting (PS) [8,9,10]. In the TS approach, it splits the received signals in the time domain, while the PS separates the signals in the power domain. Thus, the EH with AmBC prolongs the network lifetime as well as brings great convenience to mobile users. On the other hand, in order to take dynamic power supply and channel randomness of an EH system into account, we need to express the change of powers in a list of states, where the state transition can be modeled by a nonlinear function in terms of the harvested power. By modeling the change of powers at those points, the device knows when and where it should start to harvest the energy. Moreover, the harvested energy should be greater than the activation energy level in order to activate an EH circuit.

In order to support IoT and other cognitive radio operations for spectrum opportunities, enabling an integrated energy harvesting and spectrum sharing mechanism such as AmBC-enabled SWIPT should be considered. To this end, the SWIPT helps cognitive radio operation for both spectrum and energy efficiencies of the network, while the AmBC uses the ambient energy resources to support short-distance communications. The concept of the AmBC-enabled SWIPT has been introduced in [11,12]. In this paper, we consider a simple system consisting of four nodes: a source, a destination, a relay, and a secondary backscatter. The relay node-assisted AmBC receiver has the capability of SWIPT transmission in the primary link, while reflects the information to nearby receivers over the ambient RF carriers. It has been shown in [8,9,10] that deploying the PS protocol along with decode-and-forward (DF) relaying brings better performance than amplify-and-forward (AF) relaying in terms of energy harvesting and network coverage extension. A typical RF energy harvester is equipped with an antenna to capture RF signals and a rectifier to convert them into direct current for use. The amount of EH depends on several factors such as input power, RF frequency and operating distance. This involves a nonlinear relationship in the energy harvester. Thus, the conventional linear EH model may not be accurate for real measurements. With the nonlinear EH models the authors in [1,3] analyzed that a higher system capacity could be achieved compared to the conventional linear one. Following [11,12], we further provide theoretical results on the outage probability of an AmBC-enabled SWIPT DF relaying system. Specifically, the contribution of this paper is summarized as follows.

(i) We first study a wireless energy relaying system where each communication is performed in two stages: from the source to the relay and from the relay to destination. In order to deliver power and information simultaneously by the same signal, the relay is equipped with a PS mechanism to split the incoming signals in the power domain at the receiver. Besides the EH relaying, the relay also corporates with other neighboring nodes by reflecting its own information via an AmBC protocol. Motivated by the aforementioned observations, we consider two communication protocols at the relay node: the SWIPT for the primary communication and the AmBC for the secondary communication. Thus, the AmBC-enabled SWIPT relaying not only recharges the relay nodes via SWIPT, but also offers low power communication via AmBC without any extra infrastructure. We also provide details about system features along with data transmission procedures.
(ii) Next, we present a nonlinear EH model that is commonly used in energy harvesting applications. This reflects the characteristics of an EH circuit in converting the input RF power to the output direct current (DC) power for use. Under this EH model, we derive upper bounds of outage probabilities in both primary and secondary communication links, where the end-to-end transmission signal-to-noise ratio (SNR) must be larger than or equal to the required SNR threshold. Moreover, since the system adopts the PS scheme at the relay, the PS ratio is dynamically adjusted according to the channel gain at a certain communication time.
(iii) Finally, the performance of the system is analyzed through several simulation results. We show both outage probability and achievable throughput in terms of transmit power and transmission rate. We also derive energy efficiency of the proposed scheme with a nonlinear EH model and compare to the conventional one with a linear EH model [11].

The rest of the paper is organized as follows. In Section 2, we present a research background on the RF relaying system, EH modeling, and the motivation of the paper. In Section 3, we introduce a communication system model including a SWIPT-based DF in the primary transmission and an AmBC-based DF in the secondary transmission. We present our theoretical analyses on the outage probability and the achievable throughput for the given system in Section 4. In Section 5, we perform several simulations to evaluate the proposed scheme. Finally, the conclusion is given in Section 6.

## 2. Background and Motivation

### 2.1. Energy Harvesting Relaying

As presented in Section 1, in order to increase network capacity and coverage as well as to enable self-powering function, there have been lots of research work on reducing the gaps between the conventional relaying and the EH relaying networks [10,11,12,13,14,15,16,17,18,19,20,21,22,23,24,25]. The invention of RF-based wireless networks has solved pervasive issues in order to provide undisturbed continuity, connectivity and high quality-of-service. It has been shown that RF energy has several advantages over battery recharging/replacement or other energy sources [2]. This is because the RF energy harvester converts the ambient RF energy to electricity that can reduce the extra operational or maintenance cost. In fact, wireless systems exploit RF waves for information delivery, thus they also use the same RF signals for both information decoding (ID) and EH receivers with the SWIPT structure.

Typical RF energy harvester consists of three main components: an antenna, a matching network, and a rectifier [3,8]. The antenna captures the RF waves from the air and collects RF energy. The matching network adjusts overall impedance between the antenna and the rectifier to optimize the energy transfer. The rectifier is an important part that converts the energy to some voltage or current to power the RF circuit. Thus, the efficiency of the RF energy harvester is usually measured by the conversion efficiency that is defined by the ratio between the input power and the output power of the circuit. Since the amount of harvested energy from the RF sources varies with time, it leads to the uncertainty in the energy supply. Thus, it requires us to establish an accurate EH model that describes this energy uncertainty, while guaranteeing a suitable integration into the RF circuits. There are deterministic and stochastic approaches [3,13] for modeling the harvested energy. The harvested energy is assumed to be perfectly known in the deterministic approach, while it is treated as a random process in the stochastic approach. Therefore, the mismatch between the actual energy and the modeled energy may cause a performance degradation.

### 2.2. Ambient Backscatter-Enabled Relaying with SWIPT

Compared to the traditional batteries and other energy sources, ambient RF energy harvesting is sufficient for low-power devices, where the available power varies depending on communication range and propagation environment. For example, with a 1W transmitter located at a distance of 10 m, the available power density of the receiver was usually between 0.2 nW/${\mathrm{cm}}^{2}$ and 1 μW/${\mathrm{cm}}^{2}$ [14]. With these available sources, deploying EH at a relay becomes a great solution that provides a certain advantage of minimizing extra energy cost. In EH relaying networks, instead of using its own energy, a relay can absorb the harvested energy from a source to forward the signal to a destination. Therefore, backscatter-enabled relays can bring lower deployment cost and prolong lifetime compared to the traditional relays (e.g., RFID).

Based on the EH mechanism, existing approaches fall into two categories: TS relaying and PS relaying [10]. In fact, it has been shown that the PS scheme offers a better rate-energy tradeoff but it requires more complicated hardware than the TS. With a fixed relaying period *T*, if the PS is deployed an RF source transmits a signal to the relay for T/2 s, then the relay forwards the information in the next T/2 s. Since the signal is separated into two parts for the ID and the EH circuits, it is important to estimate the PS factor under certain restrictions such as maximum throughput or number of hops. With the PS in AF relaying, since the portions for the EH and the relaying are different, it leads to ineffective energy usage. For example, assuming that *T* is fixed, if at first, the relay splits a small portion of the received signal for the relaying and a large portion for the EH, it transmits a very weak signal in the next relay. On the other hand, the authors in [8] showed that with the PS scheme a DF relay had the potential to fully exploit the SWIPT and to improve relaying efficiency.

### 2.3. Motivation

We would like to summarize remaining issues of the aforementioned works [9,10,11,18,19,20] as follows. First, the conversion process of an ambient RF source into a usable electric energy can be modeled by a nonlinear input-to-output mapping with one or multiple maximum points [13]. The maximum point is usually realized from the energy profile during transitory regimes. As aforementioned in Section 1, a fully robust harvesting model may consider the nonlinear feature of the EH rectifier to reflect its uncertainty. Second, the randomness of the harvested energy leads to the randomness in the energy availability at the relay, thus affects the transmission policy [26]. For instance, SWIPT is adopted in the relay system so that the relay can utilize the EH from the ambient RF signals [12]. An AmBC-assisted SWIPT system is investigated in which full-duplex cooperation is adopted with the assumption of the existence of other secondary transmission links. It is observed that in the fading channels, the optimal PS ratio is adaptively adjusted based on the wireless channel gains in order to maximize the achievable transmission rate [27]. Then, we should investigate the nonlinear relationships between the system performance (e.g., outage probability and system throughput) versus various factors such as transmit power, energy conversion efficiency and operating distance as well as the energy-efficiency which is defined by the ratio of total achievable throughput and the total power consumption. Despite recent works, we further study, in this paper, several perspectives of the transmission performance and the system reception such as PS ratio, outage probability and energy efficiency, if a nonlinear energy harvester is used. Following [12], we present a problem formulation and provide some new results such as derivations of the optimal PS ratio and the optimal distance, the upper bounds of outage probabilities for both primary and secondary systems. We also compare the energy efficiency with the conventional EH linear model [11].

## 3. System Description

In this section, we first introduce the basic structure of a typical AmBC-enabled SWIPT relaying system which has three major operations: energy harvesting, information transmission and backscatter. Then, two important phases for simultaneous transmission are presented along with performance metrics to measure the capacity and the reliability of the system. Finally, we address some practical issues when the relay performs one or two receptions depending on the received signals from the source. Moreover, in this paper, we adopt the mathematical symbols used in [12], while adding more new ones in Table 1.

### 3.1. System Components

**Figure 1:**
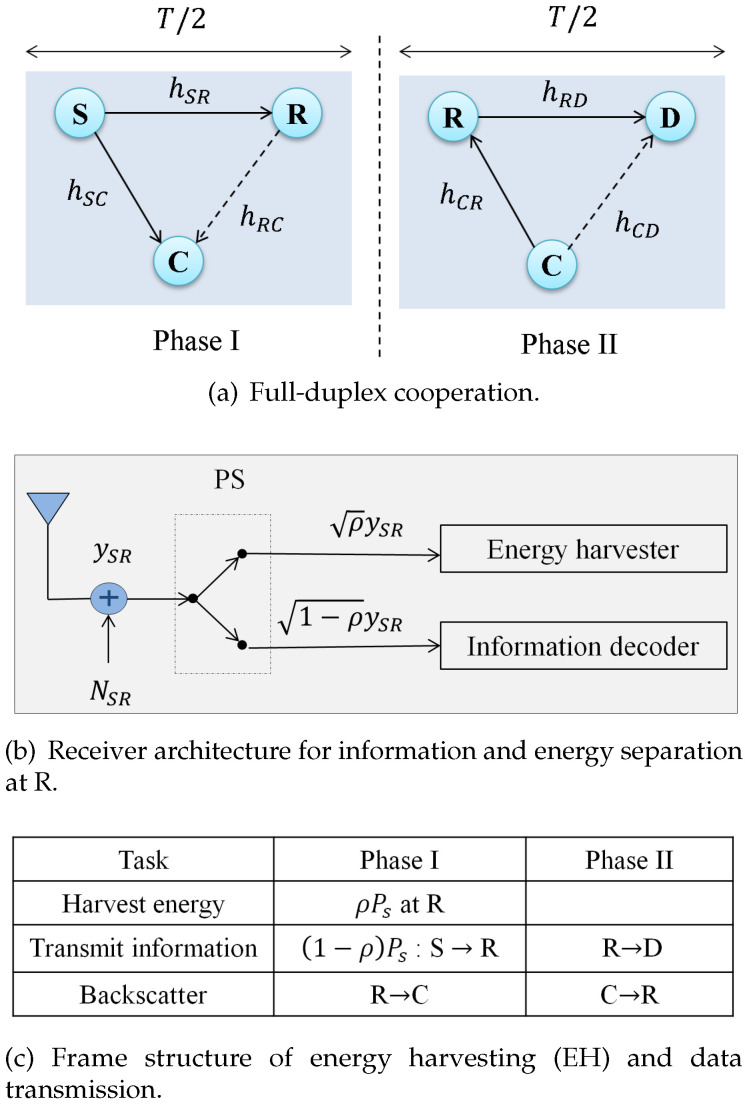
System diagram.

The proposed system has four accompanying components: primary source (S), destination (D), relay (R) and second backscatter (C) as depicted in Figure 1a. Based on [12], the following two assumptions have been made in order to ensure instantaneous spectrum opportunities on communication channels.

(A1) R is able to dynamically collaborative data transmission and energy harvesting by the antenna selection schemes.
(A2) R uses DF relaying with PS strategy with the same codebook as that of the source S, where the receiver structure for splitting information and energy at R is depicted in Figure 1b.

Here, we summarize the characteristics that each component should have. In regard to the primary communication, since we assume that R is typically energy-constrained, it exploits the SWIPT to recharge itself. In particular, R harvests energy from its received RF signals, then modulates its own information by intentionally switching its antenna coefficients to reflect the modulated signals to D. For the secondary communication, node R utilizes AmBC for the link R→C. By this way, R acts as a passive reflector to support secondary communication, which does not require specific extra power supply infrastructure. In terms of SWIPT-enabled AmBC communication system, it involves the joint activity between two phases as shown in Figure 1c. The source S communicates with R via SWIPT by dividing the received data during a time slot. Each received signal is split into two streams according to the PS ratio ρ, one for ID and the rest for EH. The rate/energy efficiency can be examined by using a version of the sum rate optimization. With the DF protocol, the R first decodes the signal for information then encodes the information again before forwarding it to D, while it backscatters it to C. Thus, it can remove the noise at the relay by performing both decoding and encoding. In summary, by integrating SWIPT and AmBC, the proposed system is suitable for both low power and low data rate scenarios.

In this paper, we assume all the squares of the channel amplitudes $|h_{ij}{|}^{2}$ (i,j∈{S,D,R,C}) are mutually independent and follow quasi-static Rayleigh fading. Mathematically, we write $|h_{ij}{|}^{2}∼R\left(\Omega_{ij}\right)$ with the probability density function (PDF) as:

(1) $$f_{|h_{ij}{|}^{2}}\left(z\right)=\frac{1}{\Omega_{ij}}e^{-z/\Omega_{ij}}U\left(z\right)$$

The rate parameter $\Omega_{ij}$ is obtained by taking the expectation operator as $\Omega_{ij}=E[|h_{ij}{|}^{2}]=\lambda_{ij}d_{ij}^{-\nu }$, where $\lambda_{ij}$ is the power of Rayleigh fading, $d_{ij}$ is the distance of the link and ν is the scale parameter. The corresponding cumulative distribution function (CDF) is given by:

(2) $$F_{|h_{ij}{|}^{2}}\left(z\right)=\left(1-e^{-z/\Omega_{ij}}\right)U\left(z\right)$$

We also use the fact that if two random variables *X* and *Y* are independent the PDF of Z=XY is given by [28]:

(3) $$f_{Z}\left(z\right)=\int_{R}\frac{1}{\left|t\right|}f_{X}\left(t\right)f_{Y}\left(\frac{z}{t}\right)dt$$

Moreover, for β≥0 and γ>0 we have:

(4) $$\int_{0}^{\infty }\exp\left(-\frac{\beta }{4x}-\gamma x\right)dx=\sqrt{\frac{\beta }{\gamma }}K_{1}\left(\beta \gamma \right)$$

where $K_{1}\left(\cdot \right)$ is the first-order modified Bessel function of the second kind [28]. The properties of this function provide some key insights to derive the outage probability in the next section.

### 3.2. Cooperative Transmission Strategy

In the above system, the information is transmitted from the source S through the relay R to the destination D in one direction. In this case, the transmission is exploited in two phases. In the first phase, S transmits the signals to R and R backscatters the information to the secondary receiver C. In the second phase, R forwards the received signal to D, while C is expected to transmit the data during a certain slot. Thus, R performs two communication protocols and two transmissions depending on the existence of the primary link from the source. Compared to the conventional scheme that separates the SWIPT [9] and the AmBC at the relay [10], the proposed scheme provides an efficient method of both data transmission and charging mechanism.

We also use the following assumptions. With this simple system, R can decode and obtain useful information from S, then forward its decoded signal only if its received SNR is greater than a certain threshold. Otherwise, R keeps silent and D decodes based on the primary transmission from S only. Second, among various relaying strategies, we only consider the DF because it performs significantly better than the AF under the same bandwidth efficiency and power consumption [2,3]. Under the DF protocol, the relay node can use its SNR to proceed the transmitted signal from the source or not by checking whether the SNR of the signal is below an acquired SNR threshold. This action can be considered as a signal quality check. Thus, it can prevent the error in the first transmission from propagating to the next transmission. In particular, we revisit the data transmission strategy in [11,12] as follows.

#### 3.2.1. Phase I-Transmission

During the phase I, the received signal and the corresponding SNR at the relay are represented by:

(5) $$y_{SR}=\sqrt{\frac{P_{s}}{d_{SR}^{m}}}h_{SR}x_{s}+N_{SR}$$

(6) $$\gamma_{R}=\frac{P_{s}{\left|h_{SR}\right|}^{2}}{d_{SR}^{m}\sigma^{2}}$$

where $N_{SR}∼N\left(0,\sigma^{2}\right)$ is the additive noise. Using the aforementioned assumptions, the relay node R splits the received signal $y_{SR}$ into ID and EH parts by using PS as follows:

- Part of $y_{SR}$ is received at the relay for information delivery. Denoting the signal is delivered to the ID circuit at R as $y_{ID}^{I}$ and its corresponding SNR during the first transmission phase as $\gamma_{R}^{I}$, we have:
  (7) $$y_{ID}^{I}=\sqrt{\frac{\left(1-\rho \right)P_{s}}{d_{SR}^{m}}}h_{SR}x_{s}+N_{SR}$$
  (8) $$\gamma_{R}^{I}=\frac{\left(1-\rho \right)P_{s}{\left|h_{SR}\right|}^{2}}{d_{SR\sigma^{2}}^{m}}$$
- Another part of $y_{SR}$ is harvested by the relay. The amount of harvested energy during this phase is defined by:
  (9) $$P_{h}=\rho \frac{P_{s}{\left|h_{SR}\right|}^{2}}{d_{SR}^{m}}$$

The achievable rate of the link S→R for the first transmission is calculated as:

(10) $$R_{s}=\frac{1}{2}\log_{2}\left(1+\gamma_{R}^{I}\right)=\frac{1}{2}\log_{2}\left(1+\frac{\left(1-\rho \right)P_{s}{\left|h_{SR}\right|}^{2}}{d_{SR}^{m}\sigma^{2}}\right)$$

Next, we present how R exploits the AmBC in the secondary communication. First, it transmits its own secondary information to C by backscattering the received signal $y_{SR}$. Denoting the signal backscattered by R as $x_{b}=\xi sy_{SR}$, the received signal at C is given by:

(11) $$y_{C}=\sqrt{\frac{P_{s}}{d_{SC}^{m}}}h_{SC}{\tilde{x}}_{s}+\xi s\left(n\right)y_{SR}$$

Here, *s* is the tag symbol, ξ∈(0,1] is the attenuation coefficient inside the relay, ${\tilde{x}}_{s}$ is the signal after decoding and $N_{RC}∼N\left(0,\sigma^{2}\right)$ is the additive noise. Similarly, we define the the received SNR at the secondary node C as:

(12) $$\gamma_{C}=\xi \frac{P_{s}|h_{SR}{|}^{2}{\left|h_{RC}\right|}^{2}}{d_{SR}^{m}d_{RC}^{m}\sigma^{2}}$$

#### 3.2.2. Phase II-Transmission

For the primary transmission, the received signal at the destination can be given by:

(13) $$y_{RD}=\sqrt{\frac{P_{r}}{d_{RD}^{m}}}h_{RD}x_{r}+I_{r}+N_{RD}$$

where $P_{r}$ is the transmit power at the relay and $N_{RD}∼N\left(0,\sigma^{2}\right)$ is the additive noise. Note that $P_{r}$ often depends on several important factors such as the amount of stored energy at R, so exhibits a nonlinear characteristic which will be considered in the next section. From Equation (13), the corresponding signal-to-interference-plus-noise ratio (SINR) at D is given by:

(14) $$\gamma_{D}=\frac{P_{r}{\left|h_{RD}\right|}^{2}}{d_{RD}^{m}\left(\sigma^{2}+I_{r}\right)}$$

The factor $I_{r}=\xi \sqrt{\frac{P_{r}}{d_{RC}^{m}d_{CD}^{m}}}{\left|h_{RD}\right|}^{2}{\left|h_{CD}\right|}^{2}$ is the ambient interference caused by the secondary receiver C. The distance $d_{CD}$ satisfies the law of cosines, i.e., $d_{CD}^{2}=d_{RC}^{2}+d_{RD}^{2}-2d_{RC}d_{RD}\cos\left(∠\left(CRD\right)\right)$. Furthermore, the secondary SNR at R in this phase is calculated as:

(15) $$\gamma_{R}^{II}=\xi \eta \frac{|h_{RC}{|}^{2}{\left|h_{CR}\right|}^{2}}{d_{SR}^{m}d_{RC}^{m}d_{CR}^{m}\sigma^{2}}(P_{s}{\left|h_{SR}\right|}^{2}-\gamma_{0}d_{SR}\sigma^{2})$$

where $|h_{SR}{|}^{2}\geq (\gamma_{0}d_{SR}^{m}\sigma^{2})/P_{s}$. Before going through the main results, we would like make the following remark on the limitations of this cooperative system.

**Remark** **1** (Loop-back self-interference issue at the relay node and potential solution). *It has been reported in [11,15,16] that when data transmission and energy harvesting at a node occur simultaneously, the loop-back self-interference problem occurs. This problem is more serious especially when the relay node adopts both SWIPT and AmBC. Note that the problem of self-interference is very common in a full-duplex transmission [16]. In this case, if we consider the TS scheme at the relay, it can still transmit data by one antenna and harvest energy from other antenna. Then, the loop-back signals provide the opportunity for harvesting more energy to EH antenna. Meanwhile, if we adopt the PS scheme at the relay in half-duplex mode, the situation becomes less serious since the data transmission and energy harvesting do not occur simultaneously. With the system model in this section, in order to mitigate the self-interference, several system architectures and cancellation techniques have been proposed within two main categories: passive suppression and active cancellation [11,16,17]. In the passive suppression approach, it can be done by suppressing the transmitted signal in the propagation domain before processing at the receiver, while in the active cancellation approach, a copy of the transmitted RF self-interference signal is generated and subtracted from the received signal in the RF domain. In terms of additional energy-efficiency gain, the authors in [15] proposed an approach for self-energy recycling, where the self-interference from data transmission was reprocessed back to the energy harvester for later use. In this paper, we are more interested in designing a complete EH system starting with a harvested model and analysis toward different system perspectives. This includes details on the optimal PS ratio and the distance between two receivers that are necessary to achieve a highly efficient SWIPT system. Thus, the investigation of self-interference cancellation techniques is beyond the current scope of this paper.*

## 4. Performance Analysis

Typical EH circuit is made up with nonlinear devices such as a rectifier, thus exhibiting a nonlinearity characteristic. The conventional works [8,9,10,11] often treat the conversion efficiency η as a constant which takes a value in the interval [0,1]. Meanwhile, the authors in [13,20,25,29,30,31,32,33] tried to capture the nonlinearity of the EH circuit, where the amount of harvested power was a function of other parameters (e.g., conversion efficiency η and harvested energy $E_{h}$) of the EH receiver. In this section, we consider the same system of [11] but different energy conversion efficiency. We observe the following. (i) η is supposed to reflect the capability of the RF-to-DC conversion circuit. Thus, it depends on the level of the input power, which is influenced by the nonlinear elements of the EH circuit. (ii) The harvested power cannot exceed the maximum capacity of energy storage. Therefore, we aim to consider the limited RF energy constraints as well as the nonlinear relationship between the input and the output. Moreover, the optimal PS ratio will be adapted to the channel condition and the harvester design (i.e., SWIPT with DF capability). Using predefined SNR thresholds in both primary and secondary transmission systems, we analyze the corresponding outage probability and achievable throughput for those systems.

### 4.1. Theory of Nonlinear Energy Harvesting

There have been considerable works on the nonlinear characteristics of EH circuits [3,16,18,19,20]. Accordingly, depending on the EH receiver architecture, two main approaches have been identified either as (i) performing the harvested power directly as a nonlinear function, or (ii) treating the conversion efficiency of the EH circuit η as a dynamic term. In this section, we refer the second approach where the conversion efficiency η is a key factor. It reflects the input–output relationship as:

(16) $$\eta =\frac{P_{\mathrm{DC}-\mathrm{out}}}{P_{\mathrm{RF}-\mathrm{in}}}$$

where $P_{\mathrm{DC}-\mathrm{out}}$ is the output DC power and $P_{\mathrm{RF}-\mathrm{in}}$ is the the input RF power. Existing practical EH circuits often exploit characteristic of quasi-concave functions [3,13,21]. One example is the logistic (sigmodal) function which has been recognized in the conventional works. In the later section, we adopt this function to represent a nonlinear phenomenon in the EH circuit sensitivity. We can also write the output power $P_{\mathrm{DC}-\mathrm{out}}$ as a function of the input power and the factor η as $P_{\mathrm{DC}-\mathrm{out}}=\eta P_{\mathrm{RF}-\mathrm{in}}$. In other words, if the input power $P_{\mathrm{RF}-\mathrm{in}}$ is fixed maximization of the efficiency is equivalent to the maximization of $P_{\mathrm{DC}-\mathrm{out}}$. Thus, one can apply tuning techniques [21,22,24] to find the related parameters of the EH circuit so that it can generate the maximum power for the best performance. Moreover, in order to characterize the sensitivity of the EH receiver, we have to consider the EH circuitry activation threshold in the energy harvesting process, while representing the peak power constraint at the EH receiver. In this section, we investigate a more general scenario that takes the nonlinearity nature of the EH circuit into account and reveal its effects on throughput/energy efficiency performance of the SWIPT-enabled AmBC system. Alternatively, the peak efficiency at an energy harvester operates according to the input power range. It has been shown in [29] that RF energy harvester can work well over a long distance at a lower input power level and high circuit sensitivity.

As illustrated in Figure 1b, in order to involve the nonlinearity of the EH circuit at R, we need to model the harvested power at the EH receiver in [20]. Thus, the harvested power $P_{r}$ is expressed as a piecewise linear EH model as:

(17) $$P_{r}=\left\{\begin{array}{cc} \eta P_{h} & \mathrm{if}\;0\leq \eta P_{h}<M\\ M & \mathrm{if}\;\eta P_{h}\geq M \end{array}\right.$$

where *M* represents the peak power constraint at the EH receiver. Note that Equation (17) can be used immediately in the next relaying transmission or stored for future use, which depends on the EH strategy for relaying [26]. From Equation (17), the CDF of $P_{r}$ is given by:

(18) $$F_{P_{r}}\left(x\right)=\left\{\begin{array}{cc} 0 & \mathrm{if}\;x<0\\ 1-e^{-x/\eta {\bar{P}}_{h}} & \mathrm{if}\;0\leq x<M\\ 1 & \mathrm{if}\;x\geq M \end{array}\right.$$

where ${\bar{P}}_{h}=\rho^{*}P_{s}\Omega_{SR}/\left(d_{SR}^{m}\sigma^{2}\right)$. Consistently, the PDF of $P_{r}$ and $\gamma_{D}$ can be obtained by taking the derivative of $F_{P_{r}}\left(x\right)$.

(19) $$\begin{array}{cc} f_{P_{r}}\left(x\right) & =\frac{1}{\eta {\bar{P}}_{h}}e^{-\frac{x}{\eta {\bar{P}}_{h}}}\left[U\left(x\right)-U\left(x-M\right)\right]+e^{-\frac{M}{\rho {\bar{P}}_{h}}}\delta \left(x-M\right) \end{array}$$

(20) $$\begin{array}{cc} f_{\gamma_{D}}\left(z\right) & =\int_{0}^{\infty }\frac{1}{x}f_{P_{r}}\left(x\right)f_{\frac{|h_{RD}{|}^{2}}{\left(\sigma^{2}+I_{r}\right)}}\left(\frac{z}{x}\right)dx=I_{1}\left(z\right)+I_{2}\left(z\right) \end{array}$$

where δ(·) is the Dirac delta function [20]. The functions $I_{1}\left(z\right)$ and $I_{2}\left(z\right)$ can be derived as:

(21) $$\begin{array}{cc} \; & I_{1}\left(z\right)=\frac{\sigma^{2}+I_{r}}{\eta \Omega_{RD}{\bar{P}}_{h}}\int_{0}^{M}\frac{1}{x}\exp\left(-\frac{x}{\eta {\bar{P}}_{h}}-\frac{\sigma^{2}+I_{r}}{\Omega_{RD}}\frac{z}{x}\right)dx \end{array}$$

(22) $$\begin{array}{cc} \; & I_{2}\left(z\right)=\frac{\sigma^{2}+I_{r}}{M\Omega_{RD}}\exp\left(-\frac{M}{\eta {\bar{P}}_{h}}-\frac{\sigma^{2}+I_{r}}{M\Omega_{RD}}z\right) \end{array}$$

**Remark** **2** (Validity and reliability of the EH model). *Generally, the ratio between the output to the input of an EH system often depends on the received power at the EH receiver and its limitation on the maximum possible harvested capacity. Conventional works on the linear EH models [18,19,20] assumed the received power at the energy harvester was constant, while in recent works [25,29,30,31,32,33], the RF energy conversion efficiency was treated as a dynamic factor according to the input power levels. For example, the harvested energy in [25] was formulated as a nonlinear normalized sigmoid function or fitted over real measurement data based on the logistic function [29]. Despite the aforementioned studies, the model in Equation (17) is still valid because of the following two reasons. First, it carries a general form that describes the relationship of input-output RF powers, i.e., the conversion efficiency of the system increases as the input power increases. Second, it was proved in [20] that Equation (17) is analytically tractable to analyzing the achievable system rate, while capturing the fluctuating character of the practical EH circuit.*

Additionally, the more important part of this work is to determine the values of related parameters and to analyze the corresponding outage probabilities, which will be given in the next section. Next, we analyze the effect of PS ratio and the distance between two adjacent receivers on the energy harvesting relaying performance under the assumptions that each node has a single antenna and is able to function in full-duplex mode. We also discuss some theoretical limits of the proposed EH model.

### 4.2. Adaptive PS Ratio and Distance Optimization

It can be shown that a sufficient PS ratio is obtained by letting the current SNR at the ID circuit $\gamma_{R}^{I}$ reach a threshold SNR $\gamma_{0}$. This threshold is a minimum SNR threshold for successful decoding at the relay. Thus, the PS ratio is selected so that $R_{s}\geq R_{0}$, which leads to the optimal $\rho^{*}$ as:

(23) $$\begin{array}{cc} \; & \frac{1}{2}\log_{2}\left(1+\frac{\left(1-\rho \right)P_{s}{\left|h_{SR}\right|}^{2}}{d_{SR}^{m}\sigma^{2}}\right)=R_{0}\Leftrightarrow \rho =1-\frac{\gamma_{0}d_{SR}^{m}\sigma^{2}}{|h_{SR}{|}^{2}P_{s}} \end{array}$$

where $\gamma_{0}=2^{2R_{0}}-1$. One sees from Equation (10) that finding optimal PS ratio $\rho^{*}$ is equivalent to maximizing the achievable rate for the best performance with a fixed power $P_{s}$. Thus, $\rho^{*}$ is expressed according to the randomness of the channel gain as:

(24) $$\rho^{*}=\max\left\{0,1-\frac{\gamma_{0}d_{SR}^{m}\sigma^{2}}{|h_{SR}{|}^{2}P_{s}}\right\}$$

When the channel condition is good (i.e., $|h_{SR}{|}^{2}\geq \gamma_{0}d_{SR}^{m}\sigma^{2}/P_{s})$, the ID circuit only needs the least required power to ensure successful decoding at the relay, while the rest of the receiver power is directed to the EH circuit. Otherwise, a fixed $\rho^{*}$ may be used in order to avoid inefficient utilization of the incoming signals between the EH circuit and the ID circuit. One suggestion is that $\rho^{*}$ can be obtained by maximizing the overall SNR from the source to the destination $\gamma_{\left\{S\to D\right\}}=\min\left\{\gamma_{R}^{I},\gamma_{D}\right\}$ as:

(25) $$\begin{array}{cc} \rho^{*} & =\arg\max_{\rho }\gamma_{\left\{S\to D\right\}} \end{array}$$

From Equations (24) and (25), in order to achieve the optimum PS, knowledge of the channel gain $h_{RD}$ is required at the beginning of the data transmission. Thus, it is only suitable for quasi-static fading channels where $h_{RD}$ changes slowly. At this point, we would like to clarify that we only present a simple model for obtaining the PS ratio, however several adaptive PS ratios for relaying have been investigated to adapt the channel conditions such as [27,33], which is out-of-scope of this paper. For simplicity, we use Equation (24) for analyzing the outage probability in the next section.

In regard to the optimal distance between the source node and the relay node, it was obtained by minimizing the outage probability as [18]:

(26) $$\begin{array}{cc} \; & \min_{d}\;\mathrm{Pr}\left(R_{s}<R_{0}\;|\;R_{s}=R_{0}\right)\\ \; & \mathrm{subject}\;\mathrm{to}\;\rho =\rho^{*},\\ \; & \;\;\;\;\;d=d_{SR},\;d\geq d_{\min} \end{array}$$

The minimum distance between two receivers is given by $d_{\min}=2n^{2}/\upsilon$, where *n* is the dimension of the receiver antenna and υ is the wavelength of the RF signal. The optimization problem in Equation (26) does not have a closed-form solution, thus the optimal distance can be solved numerically.

### 4.3. Outage Probability for the Primary Communication Link

The primary outage probability of the information rate for a predetermined threshold $\gamma_{0}$ is defined by:

(27) $$P_{out}^{\left(I\right)}=\underset{P_{o1}^{\left(I\right)}}{\underset{︸}{\mathrm{Pr}(\gamma_{R}<\gamma_{0})}}+\underset{P_{o2}^{\left(I\right)}}{\underset{︸}{\mathrm{Pr}(\gamma_{R}\geq \gamma_{0};\gamma_{D}<\gamma_{0})}}$$

First, we derive the term $P_{o1}^{\left(I\right)}$ in Equation (27) as:

(28) $$\begin{array}{cc} P_{o1}^{\left(I\right)} & =F_{\gamma_{R}}\left(\gamma_{0}\right)=\left[1-e^{-\frac{\gamma_{0}d_{SR}\sigma^{2}}{P_{s}\Omega_{SR}}}\right]U\left(\gamma_{0}\right) \end{array}$$

From Equation (17), the harvested power $P_{r}$ is calculated as:

(29) $$P_{r}=\left\{\begin{array}{cc} \frac{\eta }{d_{SR}^{m}}(P_{s}{\left|h_{SR}\right|}^{2}-\gamma_{0}d_{SR}^{m}\sigma^{2}) & \mathrm{if}\;0\geq \eta P_{h}<M\\ M & \mathrm{if}\;\eta P_{h}\geq M \end{array}\right.$$

For the second term $P_{o2}^{\left(I\right)}$, it can be represented in three parts. Each represents a different class of the origin harvested power.

(30) $$P_{o2}^{\left(I\right)}=P_{o2}^{\left(I\right)}{{|}_{\left\{0<P_{r}\leq M\right\}}\times \mathrm{Pr}\left(0<P_{r}\leq M\right)+P_{o2}^{\left(I\right)}|}_{\left\{P_{r}>M\right\}}\times \mathrm{Pr}\left(P_{r}>M\right)$$

In the following, assuming $0<P_{r}\leq M$ we express $P_{o2}^{\left(I\right)}$ in terms of the conditional probabilities as:

(31) $$\begin{array}{cc} P_{o2}^{\left(I\right)}{|}_{\left\{0<P_{r}\leq M\right\}}\; & =\mathrm{Pr}\left(P_{s}{\left|h_{SR}\right|}^{2}-\gamma_{0}d_{SR}^{m}\sigma^{2}\geq 0,|h_{RD}{|}^{2}<\frac{A}{B}+R{\left|h_{RC}\right|}^{2}{\left|h_{CD}\right|}^{2}\right) \end{array}$$

where $A=\left(\gamma_{0}d_{SR}^{m}d_{RD}^{m}\sigma^{2}\right)/\xi ,B=P_{s}{\left|h_{SR}\right|}^{2}-\gamma_{0}d_{SR}^{m}\sigma^{2}$ and $R=\xi \gamma_{0}d_{RD}^{m}/\left(d_{RC}^{m}d_{CD}^{m}\right)$. The CDF of *B* can be written as:

(32) $$\begin{array}{cc} F_{B}\left(b\right) & =F_{|h_{SR}{|}^{2}}\left(\frac{b+\gamma_{0}d_{SR}^{m}\sigma^{2}}{P_{s}}\right) \end{array}$$

which leads to $f_{B}\left(b\right)=\frac{1}{P_{s}\Omega_{SR}}\exp\left(-\frac{b+\gamma_{0}d_{SR}^{m}\sigma^{2}}{P_{s}\Omega_{SR}}\right)$. Thus, we have $P_{o2}{{|}_{\left\{0<P_{r}\leq M\right\}}\leq P_{o2}^{(I)bound}|}_{\left\{0<P_{r}\leq M\right\}}$, where

(33) $$\begin{array}{cc} P_{o2}^{(I)bound}{|}_{\left\{0<P_{r}\leq M\right\}} & =\mathrm{Pr}\left(B\geq 0,{\left|h_{RD}\right|}^{2}<\frac{A}{B}+R{\left|h_{RC}\right|}^{2}{\left|h_{CD}\right|}^{2}\right)\\ \; & =\int_{0}^{\infty }\left[1-J\right]\frac{1}{P_{s}\Omega_{SR}}e^{-\frac{\gamma_{0}d_{SR}+b}{P_{s}\Omega_{SR}}}db \end{array}$$

Here, we derive *J* as the probability that:

(34) $$\begin{array}{cc} J & =\mathrm{Pr}\left({\left|h_{RD}\right|}^{2}\geq \frac{A}{B}+R{\left|h_{RC}\right|}^{2}{\left|h_{CD}\right|}^{2}\right)\\ \; & =\int_{0}^{\infty }\;\int_{0}^{\infty }\mathrm{Pr}\left({\left|h_{RD}\right|}^{2}\geq \frac{A}{B}+Rx_{1}x_{2}\right)\times f_{|h_{RC}{|}^{2}}\left(x_{1}\right)f_{|h_{RC}{|}^{2}}\left(x_{2}\right)\;dx_{1}dx_{2}\\ \; & =\int_{0}^{\infty }\;\int_{0}^{\infty }e^{-\frac{1}{\Omega_{RD}}\left(\frac{A}{B}+Rx_{1}x_{2}\right)}f_{|h_{RC}{|}^{2}}\left(x_{1}\right)f_{|h_{RC}{|}^{2}}\left(x_{2}\right)\;dx_{1}dx_{2}\\ \; & =e^{-\frac{A}{B\Omega_{RD}}}R_{th}e^{R_{th}}E_{1}\left(R_{th}\right) \end{array}$$

where $R_{th}=\Omega_{RD}/\left(R\Omega_{RC}\Omega_{CD}\right)$ and $E_{1}\left(R_{th}\right)=\int_{R_{th}}^{\infty }e^{-t}/tdt$ is the exponential function [28] By letting $\varphi =A/\Omega_{RD}$ and $\tau =1/(P_{s}\Omega_{SR})$, the value of $P_{o2}^{(I)bound}{|}_{\left\{0<P_{r}\leq M\right\}}$ is obtained as:

(35) $$\begin{array}{cc} \; & P_{o2}^{\left(I\right)bound}{|}_{\left\{0<P_{r}\leq M\right\}}=e^{-\frac{\gamma_{0}d_{SR}\sigma^{2}}{P_{s}\Omega_{SR}}}\left(1-\frac{R_{th}e^{R_{th}}E_{1}\left(R_{th}\right)}{P_{s}\Omega_{SR}}\sqrt{\frac{\varphi }{\tau }}K_{1}\left(\sqrt{\varphi \tau }\right)\right) \end{array}$$

Similarly, in the case of $P_{r}>M$, $P_{o2}^{\left(I\right)}$ can be expressed as:

(36) $$\begin{array}{cc} P_{o2}^{\left(I\right)}{|}_{\left\{P_{r}>M\right\}} & \leq \mathrm{Pr}\left(B\geq 0,|h_{RD}{|}^{2}<\frac{\gamma_{0}d_{RD}^{m}\left(\sigma^{2}+I_{r}\right)}{M}\right)\\ \; & =\mathrm{Pr}\left(B\geq 0\right)\times \mathrm{Pr}\left(|h_{RD}{|}^{2}<\frac{\gamma_{0}d_{RD}^{m}\left(\sigma^{2}+I_{r}\right)}{M}\right) \end{array}$$

where the right hand side term of Equation (36) is given by:

$$\begin{array}{cc} \mathrm{Pr}\left(B\geq 0\right) & =e^{-\frac{\gamma_{0}d_{SR}^{m}\sigma^{2}}{P_{s}\Omega_{SR}}},\\ \mathrm{Pr}\left(|h_{RD}{|}^{2}<\frac{\gamma_{0}d_{RD}^{m}\left(\sigma^{2}+I_{r}\right)}{M}\right) & =1-e^{-\frac{\gamma_{0}d_{RD}^{m}\left(\sigma^{2}+I_{r}\right)}{M\Omega_{RD}}} \end{array}$$

Moreover, the probability of the harvested power $P_{r}$ can be obtained according to Equation (18). From Equations (28) and (36), the outage probability in Equation (27) has its upper bound given as:

(37) $$P_{out}^{\left(I\right)}\leq P_{o1}^{(I)bound}+P_{o2}^{(I)bound}$$

Consequently, we defined a lower bound of the primary achievable throughput as:

(38) $$T^{(I)bound}=\left(1-P_{out}^{\left(I\right)}\right)R_{0}$$

An equivalent expression of Equation (38) is that the primary achievable throughput is the maximum rate that can be preserved over the transmission block with a given threshold $R_{0}$. Up to now, we have given an overview of an AmBC-enabled SWIPT relay network, along with some specific aspects of receiver modeling. Working with a nonlinear EH model of Equation (17), the upper bound results have been derived in terms of the outage probability and the throughput for the primary system. In the next section, we will develop the results for the secondary communication link.

### 4.4. Outage Probability for the Secondary Communication Link

As we have mentioned earlier, the data transmission is divided into two phases. In the first phase, the source S actively transmits its message to the relay R where it is decoded. In order to decode successfully, the SNR given by Equation (6) must meet the required SNR. In the second phase, the node C transmits its decoded symbols to the destination D through backscattering at R. Thus, the end-to-end SNR at D must meet the required SNR threshold (e.g., $\gamma_{1}$) as well.

#### 4.4.1. On R→C Link during the First Transmission Phase

From Equation (12), we express the outage probability for the link R→C as:

(39) $$\begin{array}{cc} P_{RC} & =\mathrm{Pr}(\gamma_{C}<\gamma_{1})\\ \; & =\mathrm{Pr}\left(|h_{SR}{|}^{2}<\frac{\gamma_{1}}{|h_{RC}{|}^{2}}\left[\frac{\sigma^{2}d_{SR}^{m}d_{RC}^{m}}{\xi P_{s}}\right]\right)\\ \; & =\int_{0}^{\infty }\left[1-e^{-\frac{\gamma_{1}}{x\Omega_{RC}}\left(\sigma^{2}d_{SR}^{m}d_{RC}^{m}/\xi P_{s}\right)}\right]f_{|h_{SR}{|}^{2}}\left(x\right)dx\\ \; & =\frac{1}{\Omega_{SR}}\left[1-e^{-\frac{\gamma_{1}}{x\Omega_{RC}}\left(\sigma^{2}d_{SR}^{m}d_{RC}^{m}/\xi P_{s}\right)}\right]e^{-z/\Omega_{SR}}\\ \; & =1-\theta_{1}K_{1}\left(\theta_{1}\right) \end{array}$$

where $\theta_{1}=\sqrt{4\gamma_{1}\sigma^{2}d_{SR}^{m}d_{RC}^{m}/\xi P_{s}\Omega_{SR}\Omega_{RC}}$.

#### 4.4.2. On C→R Link during the Second Transmission Phase

Different from Section 4.4.1, failures of the link C→R happen when its end-to-end SNR does not meet the required SNR thresholds either in phase I or phase II. Thus, we express the outage probability of the C→R as:

(40) $$P_{CR}=\underset{P_{CR}^{\left(1\right)}}{\underset{︸}{\mathrm{Pr}(\gamma_{R}<\gamma_{0})}}+\underset{P_{CR}^{\left(2\right)}}{\underset{︸}{\mathrm{Pr}(\gamma_{R}\geq \gamma_{0};\gamma_{R}^{II}<\gamma_{1})}}$$

For given thresholds $\gamma_{0}$ and $\gamma_{1}$, we derive those probabilities as follows:

$$\begin{array}{cc} P_{CR}^{\left(1\right)} & =\mathrm{Pr}\left(\frac{P_{s}{\left|h_{SR}\right|}^{2}}{d_{SR}^{m}\sigma^{2}}<\gamma_{0}\right)=F_{\gamma_{R}}\left(\gamma_{0}\right) \end{array}$$

$$\begin{array}{cc} P_{CR}^{\left(2\right)} & =\mathrm{Pr}\left(\frac{P_{s}{\left|h_{SR}\right|}^{2}}{d_{SR}^{m}\sigma^{2}}\geq \gamma_{0};\xi \eta \frac{P_{s}|h_{SR}{|}^{2}{\left|h_{RC}\right|}^{2}}{d_{SR}^{m}d_{RC}^{m}\sigma^{2}}<\gamma_{1}\right) \end{array}$$

To calculate the outage probability $P_{CR}^{\left(2\right)}$, we first express it as:

$$\begin{array}{cc} P_{CR}^{\left(2\right)} & =\mathrm{Pr}\left(B\geq 0,|h_{SR}{|}^{2}<\left[\left(\gamma_{1}d_{SR}^{m}d_{RC}^{m}\right)\sigma^{2}\right]/\left(\eta \xi \right)\frac{1}{B|h_{RC}{|}^{2}}\right)\\ & =\mathrm{Pr}\left(B\geq 0,|h_{SR}{|}^{2}<\frac{C}{B|h_{RC}{|}^{2}}\right) \end{array}$$

where $C=[\left(\gamma_{1}d_{SR}^{m}d_{RC}^{m}\right)\sigma^{2}]/\eta \xi$. With the same techniques we have used in Section 4.3, we obtain an upper bound of the outage probability $P_{o2}^{\left(II\right)}$ as:

$$\begin{array}{cc} P_{CR}^{\left(2\right)} & \leq \int_{0}^{\infty }\left[1-\varphi \right]\frac{1}{P_{s}\Omega_{SR}}e^{-\frac{\gamma_{0}d_{SR}+b}{P_{s}\Omega_{SR}}}db \end{array}$$

Here, we have:

$$\begin{array}{cc} \varphi & =\mathrm{Pr}\left(|h_{RC}{|}^{2}-\frac{C}{B|h_{RC}{|}^{2}}\leq 0\right)=1-\mathrm{Pr}\left(|h_{RC}{|}^{2}-\frac{C}{B|h_{RC}{|}^{2}}\geq 0\right)\\ & =1-\int_{0}^{\infty }\frac{1}{\Omega_{RC}}\left(x-\frac{C}{Bx}\right)e^{-x/\Omega_{RC}}dx \end{array}$$

Thus, we obtain:

(41) $$\begin{array}{c} P_{CR}^{\left(2\right)}\leq e^{-\frac{\gamma_{0}d_{SR}^{m}\sigma^{2}}{P_{s}\Omega_{SR}}}\left[1-\frac{1}{P_{s}\Omega_{SR}}\int_{0}^{\infty }Oe^{-\frac{b}{P_{s}\Omega_{SR}}}db\right] \end{array}$$

where $O=\theta_{2}K_{1}\left(\theta_{2}\right)$ and $\theta_{2}=4C/\left(B\Omega_{CR}\Omega_{RC}\right)$. Similar to Section 4.3, the achievable throughput of the secondary system is the summation of the throughput of AmBC at the node C during the first phase and the throughput at the node R during the second phase as:

(42) $$\Gamma^{(II)bound}=\left(1-P_{RC}\right)R_{0}+\left(1-P_{CR}\right)R_{1}$$

where $R_{1}=\frac{1}{2}\log_{2}\left(1+\gamma_{1}\right)$. From the obtained theoretical results, we make the following remarks.

**Remark** **3.** *Derivation of the outage probabilities in Equations (37) and (41) was obtained by adopting step-by-step procedures. Throughout the analyses, we make the following observation. First, in the conventional communication, the harvested energy and the channel gain are unpredictable, so that the PS ratio needs to be recalculated over the energy harvesting model and the fading distribution to obtain an optimal one. The outage probability is calculated as the probability that the SNR in the desired channel is larger than the minimum required decoding SNR. Second, the maximum total achievable throughput of the system is the summation of Equations (38) and (42) as:*

(43) $$\Gamma^{\max}=\Gamma^{(I)bound}+\Gamma^{(II)bound}$$

**Remark** **4.** *It is worth noting that the relay node R adopts the PS scheme to perform the SWIPT and the AmBC protocols, while the objective is to maximize the energy efficiency. In order to see how much gain is achieved by employing the AmBC-enabled SWIPT relaying system, we calculate the total outage probability of the system without aiding the AmBC. In this case, we denote $\gamma_{R}^{0}$ and $\gamma_{D}^{0}$ as the SNR at the node R and the SINR at node D, respectively.*

(44) $$\gamma_{R}^{0}=\gamma_{R},\;\gamma_{D}^{0}=\frac{P_{r}{\left|h_{RD}\right|}^{2}}{d_{RD}^{m}\sigma^{2}}$$

*Under the same conditions, the outage probability is expressed as [11]:*

(45) $$\begin{array}{cc} P_{0} & =\mathrm{Pr}\left(\min\left\{\gamma_{R}^{0},\gamma_{D}^{0}\right\}<\gamma_{0}\right)=1-\mathrm{Pr}\left(\gamma_{R}^{0}\geq \gamma_{0},\gamma_{D}^{0}\geq \gamma_{0}\right) \end{array}$$

*Following the calculation procedures as in Section 4.3, we obtain:*

(46) $$P_{0}\leq 1-e^{-\frac{\gamma_{0}d_{SR}^{m}\sigma^{2}}{P_{s}\Omega_{SR}}}\alpha K_{1}\left(\alpha \right)$$

*where $\alpha =4\gamma_{0}d_{SR}^{m}d_{RD}^{m}\sigma^{2}\left(P_{s}\eta \Omega_{SR}\Omega_{RD}\right)$. Thus, the throughput $\Gamma_{0}$ of the conventional system is calculated by:*

(47) $$\Gamma_{0}=\left(1-P_{0}\right)R_{0}$$

## 5. Simulation Results

In this section, simulation results are presented to illustrate the effects of several factors (e.g., distance, transmit power) on the performance of derived outage probabilities in Equations (37), (39) and (40), and the corresponding achievable throughputs in Equations (38) and (42). We consider the following system parameters: desired primary transmission rate $R_{0}$ bit/s/Hz, desired secondary transmission rate $R_{1}=2R_{0}$ bit/s/Hz, primary transmit power $P_{s}=1$ W, attenuation factor inside the relay circuit ξ=0.35, peak power M=0.02 W, noise power $\sigma^{2}=0.001$ W, path loss exponent m=2.5, and distance among nodes $d_{SR}=d_{RD}$ m, $d_{RC}=d_{CR}=1.3d_{SR}$ m. Note that most of the performance parameters were adopted from [12,13,16] and optimization techniques from [16,18,19,20]. We also use the logistic function [13] to model the end-to-end conversion in a practical EH circuit, which is given by:

(48) $$P_{\mathrm{DC}-\mathrm{out}}=\frac{M\left(\frac{1}{1+e^{-a(P_{h}-b)}}-\frac{1}{1+e^{ab}}\right)}{1-\frac{1}{1+e^{ab}}}$$

where a=1500 and b=0.002 [13]. In practice, the selection of *M* depends on the maximum harvester power at the EH circuit, while two parameters a,b stay fixed and are typically obtained by a standard curve fitting method. The optimization on the performance metrics of those parameters is out-of-scope of this paper.

Moreover, in order to evaluate the energy efficiency of the proposed system, we use the ratio between the achievable sum-throughput under the unit-energy consumption, which is defined as [11]:

(49) $$E_{p}=\frac{\Gamma^{\max}}{P_{s}}$$

This factor represents the maximum energy efficiency that the system can achieve with the SNR constraints of the primary user and the secondary user. In the following, we summarize the strategy for performing numerical simulations in this section.

**Figure 2:**
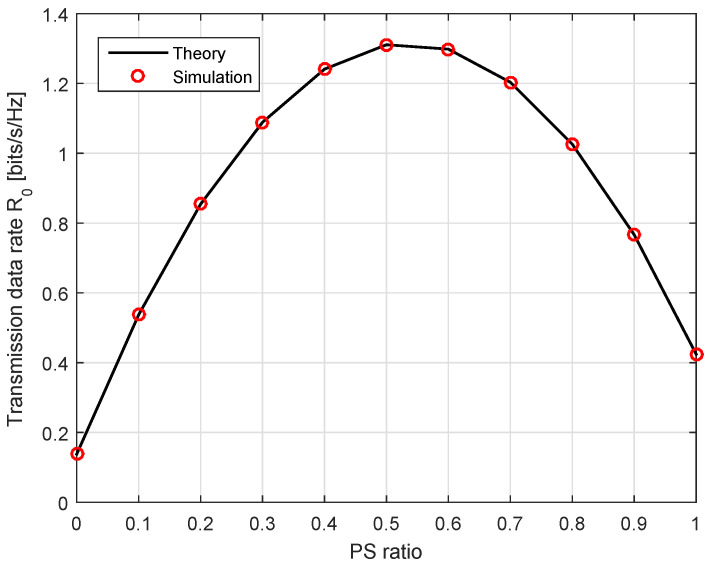
The relationship between the power splitting (PS) ratio and the transmission rate.

(i) In the context of the PS ratio discussed in Section 4.2, we keep a fixed transmit power and calculate the average optimal PS ratio by Equation (24) over a certain number of channel realizations. The relationship between $\rho^{*}$ and $R_{0}$ is graphically described in Figure 2.
(ii) Next, we investigate the impact of transmission-related parameters (e.g., transmission rates $R_{0}$ and $R_{1}$, distance between two receivers $d_{SR}$, transmit power $P_{s}$) on the outage probability and the achievable throughput. In the simulations, we depict the theory and the simulation results in the same plot, where the theory is depicted by a straight line and the simulation by bullet symbols.
(iii) We compare the energy efficiency of the conventional [11] and the proposed schemes. We also denote $E_{p}^{conv}$ and $E_{p}^{prop}$ as the energy efficiency of the system with the linear and the nonlinear EH models, respectively. For a comparison purpose, we set η=1 for the conventional scheme, while it was given as Equation (16) for our proposed scheme.

In Figure 2, we plan to obtain the ideal PS ratio in Equation (24) with various transmission rate $R_{s}$. We initially create 50 channel realizations according to the Rayleigh fading in Equation (1), then calculate the average $\rho^{*}$. We keep a fixed transmit power $P_{s}=1$ W. From the figure, we observe that the value $\rho^{*}$ tends to decrease as the transmission rate $R_{0}$ increases until it reaches the best transmission rate. It is reasonable because when ρ is small (i.e., only a small amount of energy is harvested), most signal energy is directed to the ID circuit, thereby increasing the $R_{s}$ level. Otherwise, increasing ρ leads to decreasing the transmission rate. Thus, it is important to find the optimal PS ratio in order to achieve a maximum transmission rate. According to the channel settings, we find that the optimal PS ratio which maximizes $R_{s}$ falls into the interval [0.4,0.6]. The asymmetry of the graph may be caused by the uncertain randomness of the channel gains.

**Figure 3:**
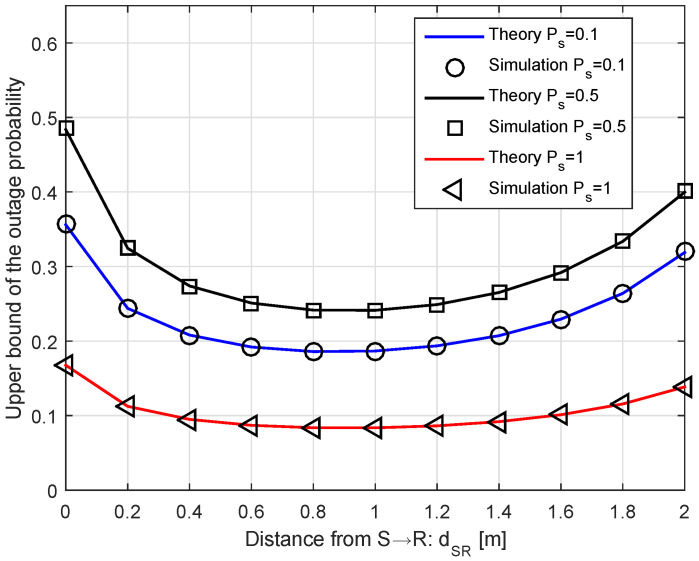
The outage probability versus the distance.

Figure 3 depicts the upper bound of the outage probability versus distance $d_{SR}$ when $R_{0}=1.25$. For each run, the theory and the simulation curves are depicted from Equation (27). In general, the primary outage probability hits the lowest point at the distance around [0.9, 1.2] m, which is known as the optimal distance between two receivers. From Equation (35), we observe that $P_{out}$ includes the Bessel function in terms of the distance $d_{SR}$ and the transmit power $P_{s}$. When $d_{SR}$ approaches the optimal distance, it enhances the amount of the harvested energy for the link S→ R, thus $P_{out}$ value decreases for a fixed transmit power $P_{s}$. After $P_{out}$ reaches the minimum point at the optimal distance $d_{SR}$, its value increases as $d_{SR}$ increases. This is because the amount of EH does not gain much, but the transmit power at the relay $P_{r}$ increases and the interference impact $I_{r}$ becomes large.

**Figure 4:**
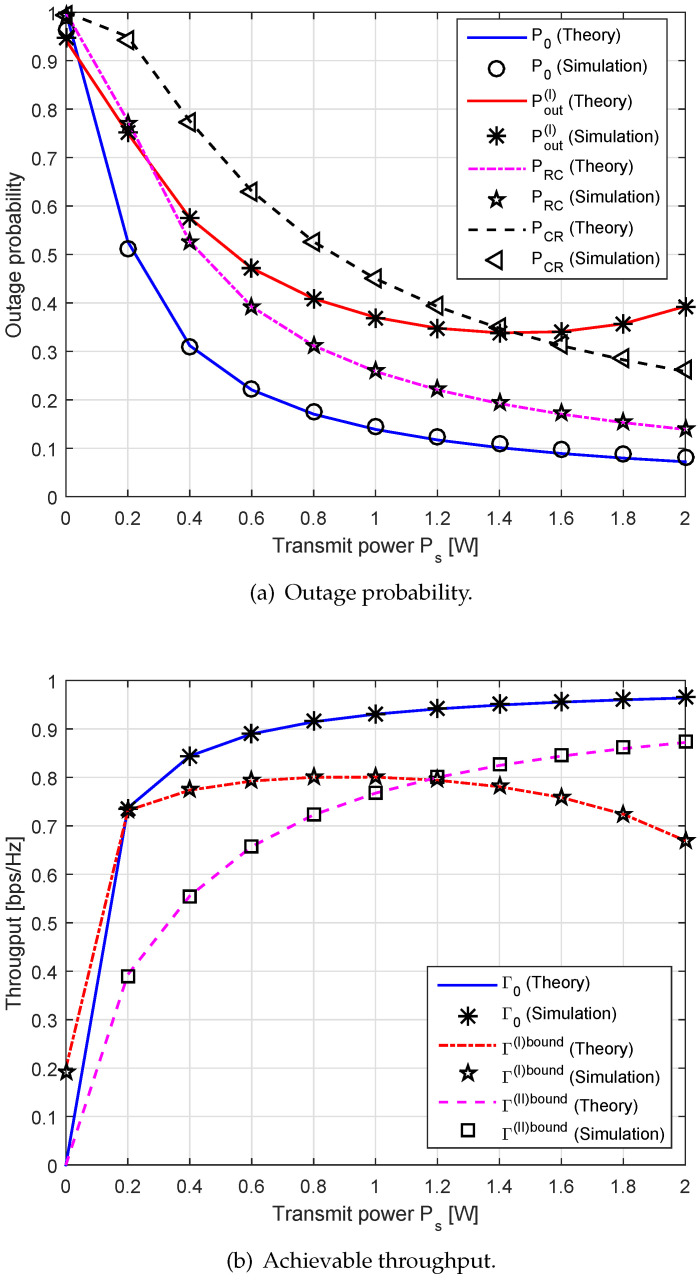
The outage probability and the throughput performance versus the transmit power $P_{s}$.

Figure 4 shows the outage probability and the throughput for different values of $P_{s}$ under $R_{0}=1$. The outage probabilities of both primary and secondary communication systems decrease as the transmit power increases. This is because when $P_{s}$ becomes large, the outage probabilities are significantly affected by SNR/SINR at the receiving nodes. Furthermore, the exponential factor increases in the outage probabilities as we increase $P_{s}$. These results are consistent with those obtained in Section 4 and with those established in [11,20]. The probability of the proposed scheme is higher than the conventional one due to the interference effects. The probability $P_{RC}$ in Equation (39) is slightly lower than other probabilities ($P_{out}^{\left(I\right)}$ and $P_{CR}$). This is because the backscatter signal from the relay to the node C via AmBC operation is the complete signal, while only a portion of the signal from S→R or from R→D is transmitted via SWIPT operation. Similarly, one observes that the achievable throughput increases as $P_{s}$ increases, which agrees with the fact that the throughput is directly proportional to the outage probability with the proportionality constant $\left(1-P_{0}\right)$. Thus, the figure also demonstrates that the higher $P_{s}$ allows better throughput performance.

**Figure 5:**
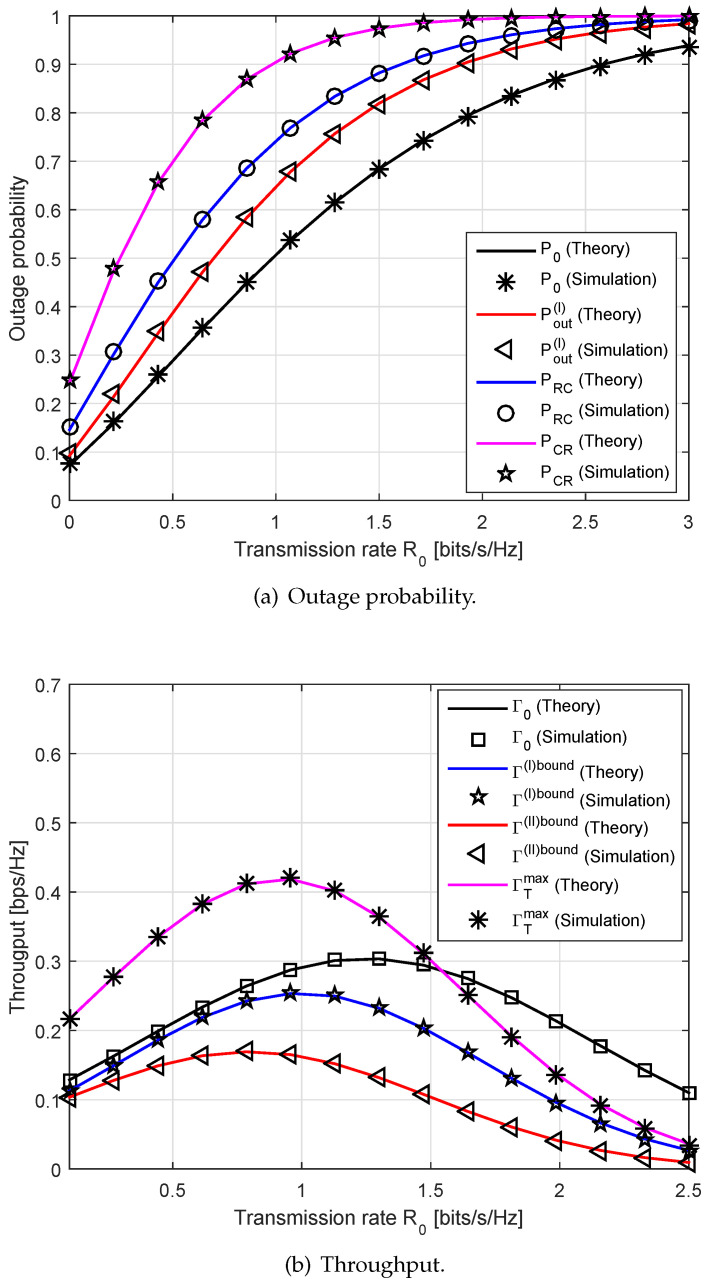
The outage probability and the throughput performance versus the transmission rate $R_{0}$.

On the other hand, Figure 5 depicts the outage probability and the achievable throughput versus the transmission rate $R_{0}$. Here, we set $d_{SR}=1$ m and $P_{s}=1$ W. We observe that the outage probability achieves low values at small values of $R_{0}$, while the throughput achieves the high values at those points. It is easy to see that the SNR/SINR thresholds $\gamma_{0}$ and $\gamma_{1}$ tend to go up as $R_{0}$ increases. Thus, the higher value of the transmission rate $R_{0}$ leads to higher outage probabilities at the receiving nodes. The total throughput of the proposed scheme is higher than that of the conventional one. Since the secondary communication causes the interference to the primary communication, it decreases the achievable primary throughput, but improves the overall system throughput. This phenomenon is well known as the sensing-throughput trade-off in typical cognitive radios [33].

**Remark** **5.** An important observation from Figure 2, Figure 3, Figure 4 and Figure 5 is that an enhancement on the system throughput not only depends on the relational distance, but also on several factors such as the PS ratio, the transmit power, and the transmission rate due to the harvested energy information. We also notice that the behavior of the outage probability curves gives very useful insight into how the optimal PS ratios for the EH should be chosen. This gives us more choices for $\rho^{*}$ according to channel conditions. Additional, the success of AmBC-assisted SWIPT protocol at the relay is affected by channel state information at all nodes as well as the RF power source. Thus, the investigation on EH capability is required for close-to-optimum performance.

**Figure 6:**
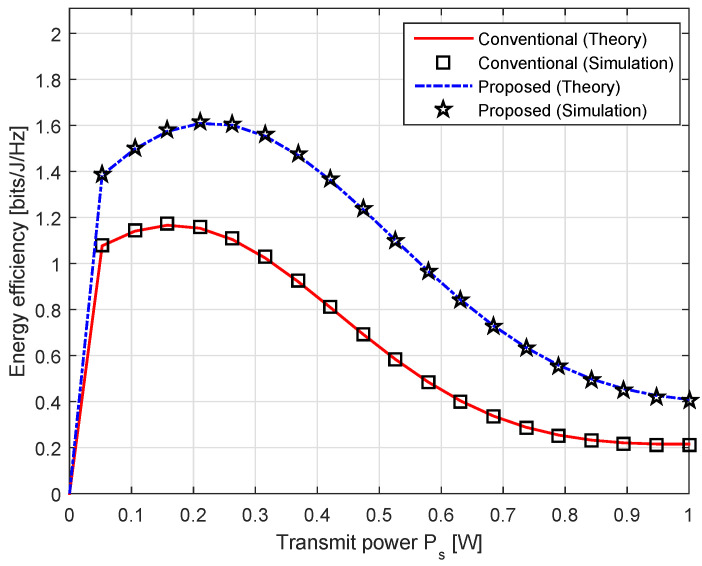
The energy efficiency with the conventional linear and the proposed nonlinear EH models.

The energy efficiency curves according to the transmit power $P_{s}$ are illustrated in Figure 6. Note that Equation (49) were obtained by averaging over different channel realizations. Thus, the energy efficiency curves exhibit relatively stable characteristic in the performance. In general, the energy efficiency of our proposed scheme is higher than the conventional one as $P_{s}$ increases when $\rho^{*}$ is obtained. The maximum energy efficiency is 1.1659 at $P_{s}=0.1579$ W in the conventional scheme, while it is 1.609 at $P_{s}=0.2105$ W in the proposed scheme with the nonlinear EH model. This also indicates that the energy efficiency is restricted by fixed parameter values. As we have explained earlier, the achievable throughput does not gain much as $P_{s}$ becomes large, leading to low energy efficiency performance.

## 6. Conclusions

In this paper, we study recent results of SWIPT and AmBC protocols, then propose a hybrid system that combines both protocols in RF-based relaying network. We explore the nonlinearity in the circuit and design suitable mathematical modeling for energy harvesting. In particular, working with a practical expression of a harvester, we have expressed the harvested power in terms of nonlinear energy conversion efficiency along with its corresponding distribution. Based on this EH model, we have further derived and analyzed the outage probability and the achievable throughput of both primary and secondary communication systems. From the simulations, the main technical results show their performance in terms of the optimal PS ratio, the distance between the source and the relay, and the transmission rate threshold. We show that our proposed scheme achieves higher overall throughput and energy efficiency than the conventional ones.

We would like to point out that it appears to be no one-fits-all EH mathematical model. The nonlinear EH function considered in this paper is only preferable in specific conditions. Thus, a suitable EH model for a particular system should be selected according to the type of excitation, the variance of RF frequency, the environment characteristics, etc. It is envisioned that, with further investigation on the EH techniques, the concept of AmBC-enabled SWIPT relaying will approach practical deployment in IoT applications. In the future, we will investigate the optimization of the transmission strategy where the energy source is modeled as a random process, and further solve the mismatch problem between the modeled energy and the actual energy in practice. 

## Figures and Tables

**Table 1:** List of mathematical symbols used in the paper.

Notation	Definition
S, D, R, C	Primary source, destination, relay, second backscatter nodes, respectively
$E_{h}$	Amount of harvested energy at the relay
E(X) and Var(X)	Mean and variance of a random variable *X*
Pr(·)	Probability of a parameter based on a particular dataset
$f_{X}\left(x\right)$ and $F_{X}\left(x\right)$	PDF and CDF of a random variable *X*, respectively
$N(\mu ,\sigma^{2})$	Normal distribution with mean μ and variance $\sigma^{2}$
$X∼N(\mu ,\sigma^{2})$	Random variable *X* follows $N(\mu ,\sigma^{2})$ with the PDF $f_{X}\left(x\right)=\frac{1}{\sqrt{2\pi }\sigma }e^{-{\left(x-\mu \right)}^{2}/2\sigma^{2}}$
R(ω)	Rayleigh distribution with rate parameter ω
X∼R(ω)	*X* follows a Rayleigh distribution with the PDF $f_{X}\left(x\right)=\frac{1}{\omega }e^{-x/\omega }U\left(x\right)$
δ(·)	Dirac delta function

## Abbreviations

The following abbreviations are used in this manuscript:

AmBC	Ambient backscatter communication
AF	Amplify-and-forward
BC	backscatter communication
CDF	Cumulative distribution function
DC	Direct current
DF	Decode-and-forward
EH	Energy harvesting
ID	Information decoding
IoT	Internet of things
PDF	Probability density function
PS	Power splitting
RF	Radio frequency
RFID	Radio frequency identification
SINR	Signal-to-interference-plus-noise ratio
SNR	Signal-to-noise ratio
SWIPT	Simultaneous information and power transfer
TS	Time switching
